# Fgf21 Impairs Adipocyte Insulin Sensitivity in Mice Fed a Low-Carbohydrate, High-Fat Ketogenic Diet

**DOI:** 10.1371/journal.pone.0069330

**Published:** 2013-07-09

**Authors:** Yusuke Murata, Kyoji Nishio, Takayuki Mochiyama, Morichika Konishi, Masaya Shimada, Hiroya Ohta, Nobuyuki Itoh

**Affiliations:** 1 Department of Genetic Biochemistry, Kyoto University Graduate School of Pharmaceutical Sciences, Sakyo, Kyoto, Japan; 2 Department of Microbial Chemistry, Kobe Pharmaceutical University, Motoyamakita-machi, Kobe, Hyogo, Japan; University of Cordoba, Spain

## Abstract

**Background:**

A low-carbohydrate, high-fat ketogenic diet (KD) induces hepatic ketogenesis and is believed to affect energy metabolism in mice. As hepatic *Fgf21* expression was markedly induced in mice fed KD, we examined the effects of KD feeding on metabolism and the roles of Fgf21 in metabolism in mice fed KD using *Fgf21* knockout mice.

**Methodology/Principal Findings:**

We examined C57BL/6 mice fed KD for 6 or 14 days. Blood β-hydroxybutyrate levels were greatly increased at 6 days, indicating that hepatic ketogenesis was induced effectively by KD feeding for 6 days. KD feeding for 6 and 14 days impaired glucose tolerance and insulin sensitivity, although it did not affect body weight, blood NEFA, and triglyceride levels. Hepatic *Fgf21* expression and blood Fgf21 levels were markedly increased in mice fed KD for 6 days. Blood β-hydroxybutyrate levels in the knockout mice fed KD for 6 days were comparable to those in wild-type mice fed KD, indicating that Fgf21 is not required for ketogenesis. However, the impaired glucose tolerance and insulin sensitivity caused by KD feeding were improved in the knockout mice. Insulin-stimulated Akt phosphorylation was significantly decreased in the white adipose tissue in wild-type mice fed KD compared with those fed normal chow, but not in the muscle and liver. Its phosphorylation in the white adipose tissue was significantly increased in the knockout mice fed KD compared with wild-type mice fed KD. In contrast, hepatic gluconeogenic gene expression in *Fgf21* knockout mice fed KD was comparable to those in the wild-type mice fed KD.

**Conclusions/Significance:**

The present findings indicate that KD feeding impairs insulin sensitivity in mice due to insulin resistance in white adipose tissue. In addition, our findings indicate that Fgf21 induced to express by KD is a negative regulator of adipocyte insulin sensitivity in adaptation to a low-carbohydrate malnutritional state.

## Introduction

Fibroblast growth factors (Fgfs) are polypeptides with diverse functions in development and metabolism. The human/mouse *Fgf* gene family comprises twenty-two members [1], [2]. Although *Fgf21* was originally identified in mouse embryos, it is predominantly expressed in the liver in adults [3]. Later, Fgf21 was also identified as a stimulator of glucose uptake in cultured adipocytes [4]. Fgf21 along with Fgf15/19 and Fgf23 are members of the endocrine Fgf subfamily. Fgf21 potentially acts as a metabolic regulator in an endocrine manner [5], [6].

Hepatic *Fgf21* expression, ketogenesis, and steatosis are greatly induced in mice fasted for 24 h [7], [8]. In *Fgf21* transgenic mice, Fgf21 acts as an inducer for hepatic ketogenesis and steatosis and for adipocyte lipolysis [7]. In Fgf21 protein-administered mice, Fgf21 also acts directly on the liver to modulate hepatic metabolism [9]. These effects are pharmacological effects of Fgf21. In contrast, blood β-hydroxybutyrate and hepatic triglyceride levels in *Fgf21* knockout mice fasted for 24 hours were comparable to those in fasted wild-type mice [8], [10], [11]. These results indicate that Fgf21 is not physiologically required for hepatic ketogenesis and steatosis induced by fasting [8]. However, blood non-esterified fatty acid (NEFA) levels and adipocyte lipolysis were significantly increased in *Fgf21* knockout mice fasted for 24 hours, indicating that Fgf21 acts as a negative regulator of adipocyte lipolysis in fasted mice [8]. These results indicate that the physiological roles of Fgf21 are different from its pharmacological effects.

A low-carbohydrate, high-fat ketogenic diet (KD), which mimics the metabolic conditions of long-term starvation, induces a unique metabolic state [12], [13]. Hepatic *Fgf21* expression and ketogenesis are greatly induced by KD feeding for at least 3 days [14]. These results suggest that KD feeding for several days markedly affects energy metabolism in mice and Fgf21 is potentially involved in the changes of metabolic state induced by KD feeding. Recently, we have found that KD feeding for 5 days significantly induced torpor, the controlled lowering of body temperature and physical activity, which is an adaptation that various mammals use to cope with periods of low food availability [15], [16]. Torpor induced by KD feeding was significantly attenuated in *Fgf21* knockout mice, indicating Fgf21 to be involved [16]. In addition, Badman reported that adenoviral knockdown of hepatic *Fgf21* expression in mice fed KD for 4 days caused reduced blood β-hydroxybutyrate levels and hepatic steatosis [14]. These results suggest that further studies using *Fgf21* knockout mice fed KD will contribute to an understanding of the metabolic roles of Fgf21.

In the present study, we examined the effects of KD feeding on metabolism and the roles of Fgf21 in metabolism in mice fed KD using a *Fgf21* knockout line. The present findings indicate that insulin sensitivity was significantly impaired in mice fed KD for several days and Fgf21 induced to express by KD is a negative regulator of adipocyte insulin sensitivity in adaptation to a low-carbohydrate malnutritional state.

## Materials and Methods

### 2.1 Animal Experiments

All mice were maintained in a light-controlled room (lights on from 0800 to 2000 h). 3-month-old C57BL/6 mice and *Fgf21* knockout mice were allowed free access to normal chow (MF; 3.6 kcal/g, 12% kcal fat, source: soybean; Oriental Yeast, Japan) or KD (TD.96355; 6.7 kcal/g, 15.3% protein, 0.6% carbohydrate, and 67.4% fat (wt/wt); Harlan, USA) for indicated periods. The experiments were performed using male mice. All mice were sacrificed to obtain tissues and blood samples at 1200 h. *Fgf21* knockout mice were generated as described [8] and maintained on a C57BL/6 background. All animal studies were conducted in accordance with International Guiding Principles for Biomedical Research Involving Animals, and approved by the Animal Research Committee of Kyoto University Pharmaceutical Sciences.

### 2.2 Blood Parameter Analysis

Blood samples were obtained from the mice. Plasma Fgf21 levels were measured using a mouse FGF-21 Quantikine ELISA Kit (R&D systems). Blood glucose levels were measured with a Glutest R kit (SANWA KAGAKU, Japan). Plasma triglyceride, non-esterified fatty acid (NEFA), and β-hydroxybutyrate levels were measured using triglyceride E-Test (WAKO, Japan), NEFA C-Test (WAKO), and ketone Test B (SANWA KAGAKU) kits, respectively. Plasma insulin, glucagon, growth hormone, and cortisol levels were measured using mouse Insulin ELISA (Morinaga, Japan), Gluagon ELISA (WAKO, Japan), Rat/Mouse Growth Hormone ELISA (Merck Millipore) Kits, and Cortisol EIA kit (Cayman Chemical Company) respectively.

### 2.3 Reverse Transcription-quantitative Polymerase Chain Reaction Analysis

Total RNA was extracted from mouse tissues using an RNeasy mini kit (Qiagen). cDNA was synthesized from the RNA (1 µg) as a template in a reaction mixture containing moloney murine leukemia virus reverse transcriptase (Gibco BRL) and a random hexadeoxynucleotide primer (Takara, Japan). Reverse transcription-quantitative polymerase chain reaction (RT-qPCR) was performed on a Thermal Cycler Dice (Takara), using SYBR Premix Ex Taq 2 (Takara). *18S* rRNA levels were used as an internal control.

### 2.4 Glucose Tolerance Test and Insulin Tolerance Test

The glucose tolerance test and insulin tolerance test were performed by intraperitoneal injection of glucose (2 mg/g) or human regular insulin (0.75 units/kg) (MP Biomedicals) after a 6-h fast [17]. Blood samples were taken at different time points from a tail vein. Blood glucose levels and plasma insulin levels were measured as described above.

### 2.5 Insulin-stimulated Akt Phosphorylation

Mice were intraperitoneally injected with insulin (1.5 units/kg). Twenty minutes later, tissues were removed and frozen in liquid nitrogen. The frozen tissue was homogenized in ice-cold lysis buffer (50 mM Tris-HCl, pH8.0, 1% Triton X-100, 2 mM EGTA, 10 mM EDTA, 100 mM NaF, 1 mM Na_4_P_2_O_7_, 2 mM Na_3_VO_4_, 100 µg/ml of phenylmethylsulfonyl fluoride, and 10 µl/ml of Proteinase Inhibitor Cocktail (P8340, Sigma-Aldrich). After homogenization, the homogenate was centrifuged at 13,000×g for 30 minutes at 4°C. The protein concentrations in the homogenate were determined using a Bio-Rad protein assay kit with BSA as a standard. The homogenate (50 µg protein each) was separated by SDS-polyacrylamide gel (12.5%) electrophoresis under reducing conditions and transferred onto a nitrocellulose membrane (Hybond-ECL, GE Healthcare Life Sciences). The protein on the membrane was detected using a rabbit anti-Akt antibody (Cell Signaling) (1∶1000) and anti-phosphoSerine-473 Akt antibody (Cell Signaling) (1∶1000) as the primary antibody in PBS containing 0.1% Tween 20 and a horseradish peroxidase-conjugated goat anti-rabbit IgG antibody (Vector Laboratories, Inc.) (1∶1000) as the secondary antibody in PBS containing 0.1% Tween 20. Immunoblots were quantified with Image J Software (National Institutes of Health, Bethesda, MD ).

### 2.6 Statistical Analysis

Data are expressed as means ± SEMs. The statistical significance of differences in mean values was assessed with Student’s or Welch’s *t*-test. *P*<0.05 was considered statistically significant.

## Results

### 3.1 Metabolic Effects of KD Feeding

**Figure 1:**
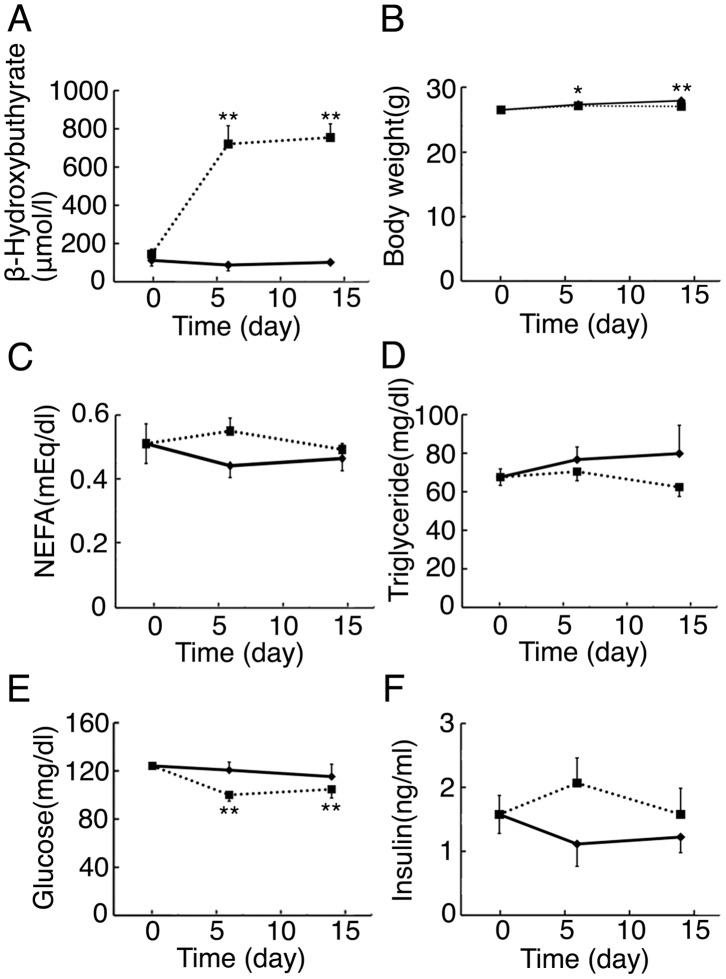
Body weights and blood parameters in mice fed KD. (A) Blood β-hydroxybutyrate levels in 3-month-old mice fed KD for 6 days or 14 days, or NC (n = 3–8 per group; **, *P*<0.01 vs. day 0). (B) Body weights in 3-month-old mice fed KD for 6 days or 14 days, or NC (n = 5–28per group; *, *P*<0.05; **, *P*<0.01 vs. day 0). (C–F) Blood NEFA, triglyceride, glucose, and insulin levels in 3-month-old mice fed KD for 6 days or 14 days, or NC (n = 4–12 per group; **, *P*<0.01 vs. day 0).

We examined C57BL/6 mice fed normal chow (NC) or KD for 6 or 14 days to elucidate the metabolic effects of KD feeding. At first, blood β-hydroxybutyrate levels in mice fed KD were examined (Figure 1A). Blood β-hydroxybutyrate levels were almost unchanged by NC feeding until 14 days. In contrast, blood β-hydroxybutyrate levels were greatly increased in m ice fed KD for 6 days, compared with mice fed normal chow (NC). The blood β-hydroxybutyrate levels in mice fed KD for 14 days were comparable to those for 6 days, indicating that hepatic ketogenesis was already induced effectively by KD feeding for 6 days. We also examined body weights and blood parameters of mice fed NC or KD. The body weights were gradually increased in mice fed NC until 14 days (Figure 1B). Until 14 days, the body weights in mice fed KD were essentially comparable to those in mice fed NC. The blood NEFA and triglyceride levels were also unchanged in mice fed NC or KD until 14 days (Figure 1C, 1D). The blood glucose levels were essentially unchanged by NC feeding until 14 days (Figure 1E). In contrast, the blood glucose levels were significantly decreased by KD feeding. The blood glucose levels in mice fed KD for 6 days were significantly lower than those in mice fed NC for 6 days (*P*<0.05). This decrease in blood glucose levels in mice fed KD suggests that blood insulin levels are decreased in the mice fed KD. But blood insulin levels in mice was not decreased but tended to be increased in mice fed KD for 6 days, compared with those of mice fed NC for 6 days (*P* = 0.12). The insulin levels in mice fed KD for 14 days were not lower than those in mice fed NC for 14 days (Figure 1F).

### 3.2 Glucose Tolerance and Insulin Sensitivity in Wild-type Mice Fed KD

**Figure 2:**
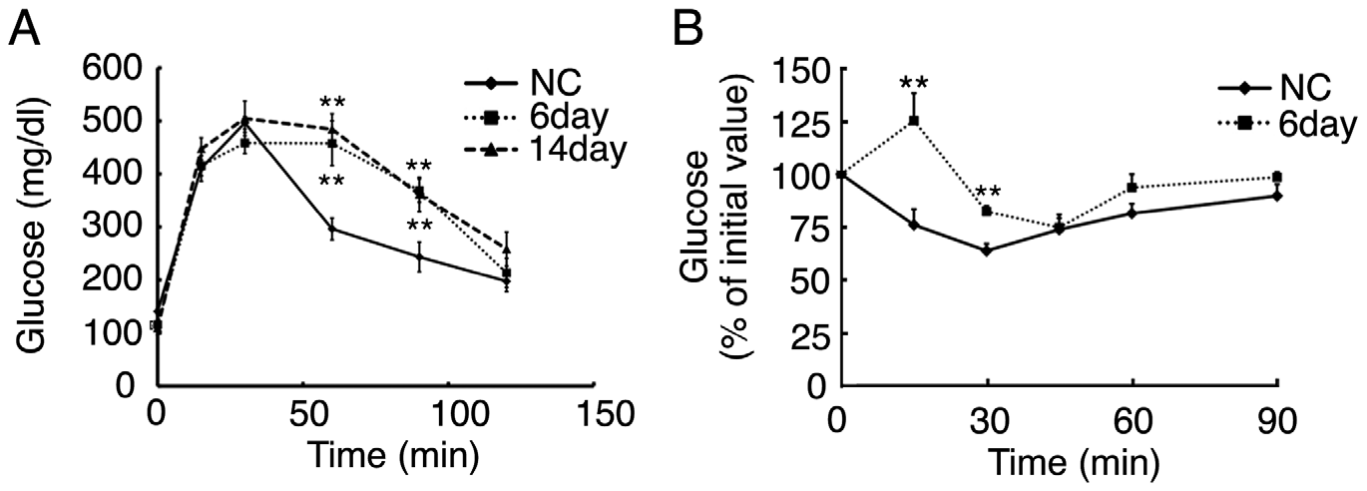
Glucose tolerance and insulin tolerance tests of mice fed KD. (A) A glucose tolerance test (GTT) of 3-month-old mice fed KD for 6 days or 14 days, or NC (n = 8–11 per group; **, *P*<0.01 vs. mice fed NC at the same timepoint). (B) An insulin tolerance test of mice fed KD for 6 days or 14 days, or NC (n = 4–7 per group; **, *P*<0.01 vs. mice fed NC at the same timepoint).

Although blood glucose levels were significantly decreased in mice fed KD, blood insulin levels were not lower than those in mice fed NC. Therefore, we examined glucose metabolism in mice fed NC or KD for 6 or 14 days using the intraperitoneal glucose tolerance test (GTT). In mice fed KD for 6 days, glucose levels remained high compared with those in mice fed NC at 60 or 90 minutes after the glucose loading (Figure 2A). The glucose excursion in response to glucose loading during the GTT in mice fed KD for 14 days was similar to that in the mice fed KD for 6 days. These results suggested that insulin sensitivity might be impaired by KD feeding for 6 days. Therefore, we next examined insulin sensitivity in mice fed KD for 6 days using insulin tolerance test (ITT). During the ITT, glucose levels were significantly higher in mice fed KD than those fed NC at 15 and 30 minutes after the insulin loading (Figure 2B). Therefore, mice fed KD for 6 days already exhibited impaired insulin sensitivity, resulting in glucose intolerance potentially.

### 3.3 Hepatic Fgf21 Expression and Blood Fgf21 Levels in Wild-type Mice Fed KD

**Figure 3:**
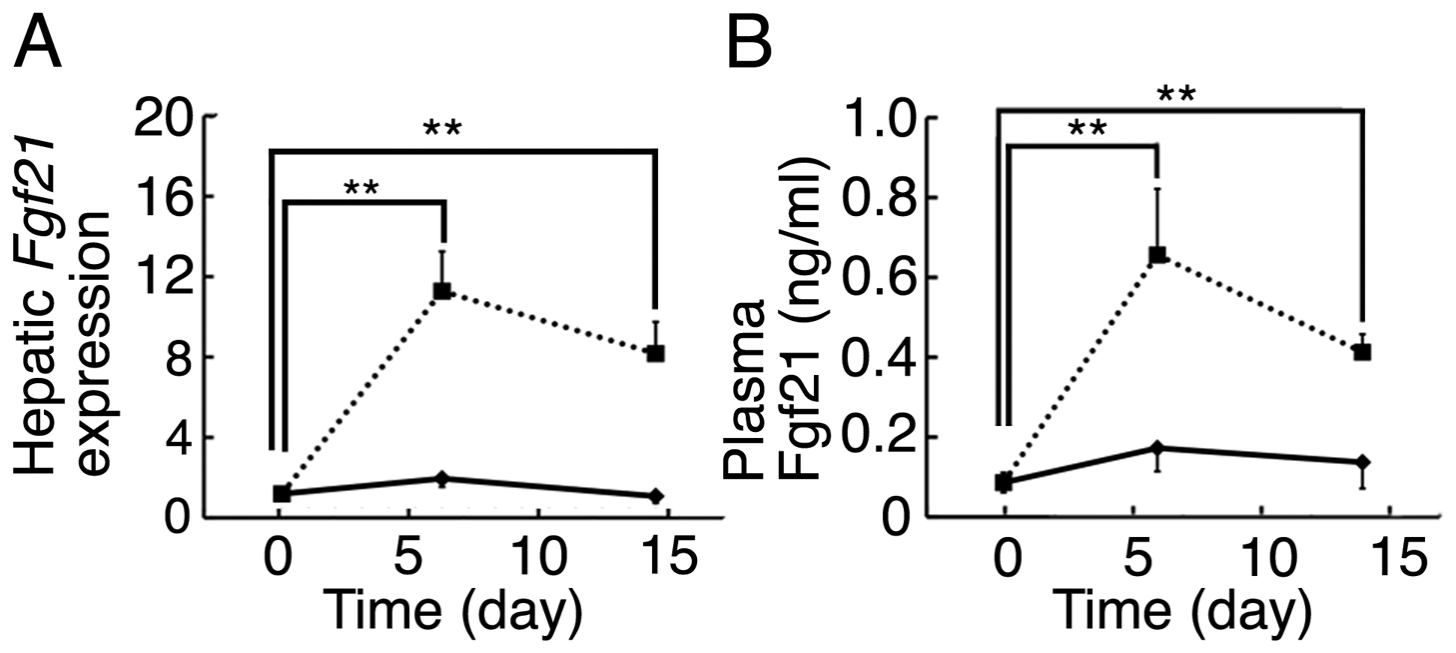
Hepatic *Fgf21* expression and blood Fgf21 levels of mice fed KD. (A) Hepatic *Fgf21* expression of 3-month-old mice fed KD for 6 days or 14 days, or NC determined by RT-qPCR (n = 4–8 per group; **, *P*<0.01 vs. day 0). (B) Blood Fgf21 levels of 3-month-old mice fed KD for 6 days or 14 days, or NC determined by ELISA (n = 5–7 per group; **, *P*<0.01 vs. day 0).

We examined hepatic *Fgf21* expression and blood Fgf21 levels in 3-month-old mice fed NC or KD by RT-qPCR. Although hepatic *Fgf21* expression was unchanged by NC feeding until 14 days, the expression was markedly increased in the mice fed KD (Figure 3A). The hepatic *Fgf21* expression induced by KD for 6 days was comparable to that induced by fasting (data not shown). The hepatic *Fgf21* expression in mice fed KD for 14 days was comparable to that in the mice fed KD for 6 days. Blood Fgf21 levels changed in parallel with the changes of hepatic *Fgf21* expression caused by KD feeding (Figure 3B).

### 3.4 Apparently Normal Phenotypes of Fgf21 Knockout Mice Fed KD for 6 Days

**Figure 4:**
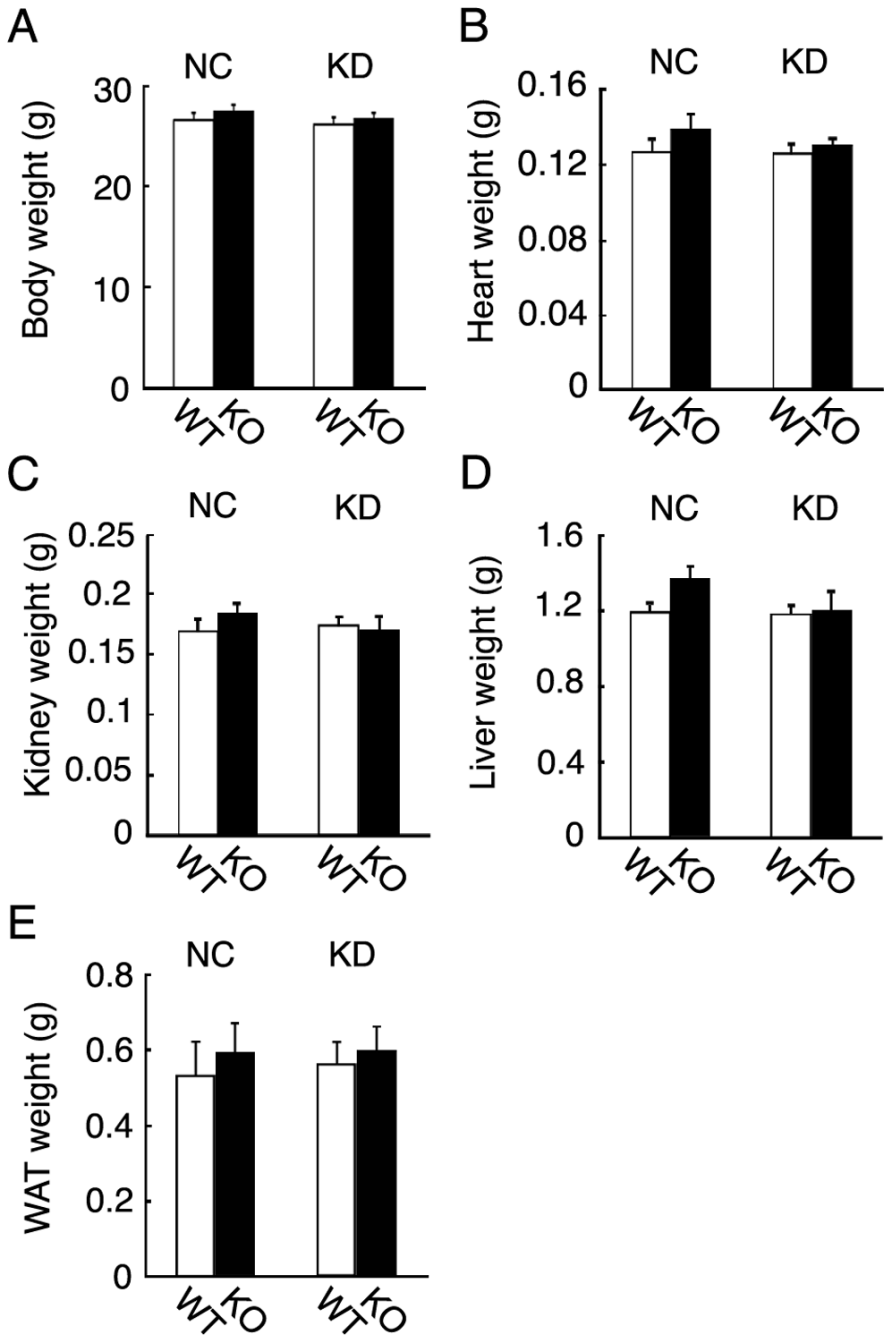
Body and tissue weights of wild-type and *Fgf21* knockout mice fed NC or KD. (A) Body weights of 3-month-old wild-type (WT) and *Fgf21* knockout (KO) mice fed NC or KD for 6 days (n = 7–8 mice per group). (B–E) The heart, kidney, liver, and subcutaneous WAT weights of 3-month-old wild-type (WT) and *Fgf21* knockout (KO) mice fed NC or KD for 6 days (n = 7–8 mice per group).

Our results indicated that KD feeding for 6 days impaired insulin sensitivity and increased blood Fgf21 levels, suggesting that Fgf21 has physiological roles in the insulin insensitivity induced by KD feeding for 6 days. Therefore, we examined the potential roles of Fgf21 using *Fgf21* knockout mice fed KD for 6 days. Both wild-type and *Fgf21* knockout mice fed KD for 6 days were apparently normal (data not shown). The body weights of the knockout mice were comparable to those of the wild-type mice (Figure 4A). We also examined tissue weights of wild-type and *Fgf21* knockout mice fed NC or KD. Heart, kidney, liver, and subcutaneous white adipose tissue weights of *Fgf21* knockout mice fed NC or KD were comparable to those of wild-type mice (Figure 4B–E).

### 3.5 Blood β-hydroxybutyrate Levels and Hepatic Ketogenesis in Fgf21 Knockout Mice Fed KD

**Figure 5:**
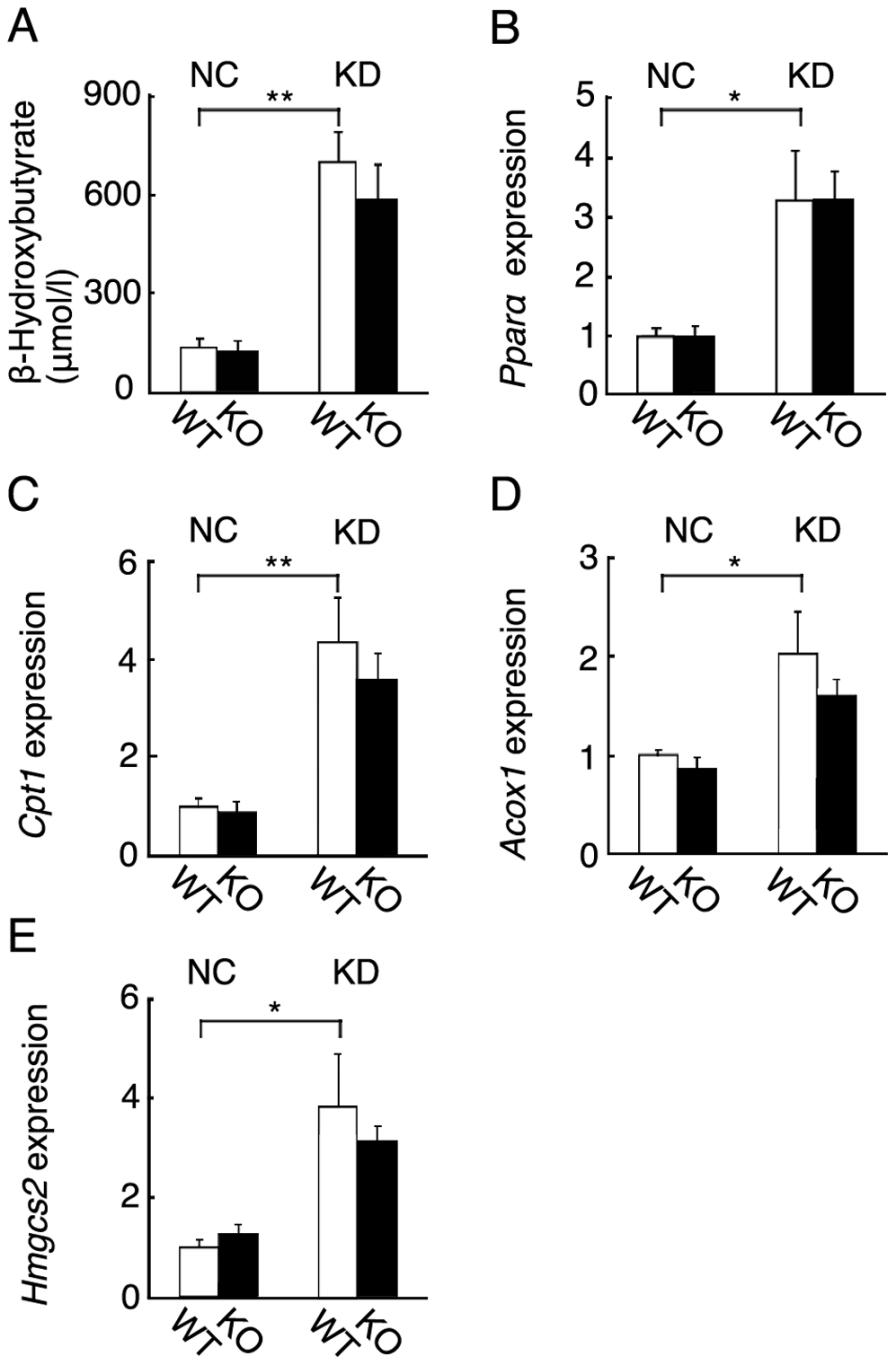
Hepatic ketogenesis of wild-type and *Fgf21* knockout mice. (A) Blood β-hydroxybutyrate levels in 3-month-old wild-type (WT) and *Fgf21* knockout (KO) mice fed NC or KD for 6 days. (n = 12–14 mice per group; **, *P*<0.01). (B–E) Hepatic expression of genes involved in ketogenesis including *Ppara*, *Cpt1*, *Acox1*, and *Hmgcs2* in wild-type (WT) and *Fgf21* knockout (KO) mice fed NC or KD for 6 days. (n = 7–9 mice per group; *, *P*<0.05; **, *P*<0.01).

We examined blood β-hydroxybutyrate levels and hepatic ketogenesis in wild-type and *Fgf21* knockout mice fed KD for 6 days. Blood β-hydroxybutyrate levels were greatly increased in both groups of mice. Blood β-hydroxybutyrate levels in the knockout mice were comparable to those in the wild-type mice (Figure 5A). We also examined the hepatic expression of genes involved in ketogenesis including *Peroxisome proliferator-activated receptor-α* (*Ppara*), *Carnitine palmitoyltransferase I* (*Cpt1*), *acyl-CoA oxidase 1* (*Acox1*), and *3-Hydroxy-3-methylglutaryl-CoA synthase 2 (mitochondrial)* (*Hmgcs2*) by RT-qPCR. Their expression levels were markedly increased in both wild-type and *Fgf21* knockout mice fed KD (Figure 5B–E). However, the levels in the knockout mice were essentially comparable to those in the wild-type mice.

### 3.6 Blood Glucose, Insulin, and Glucagon Levels in Fgf21 Knockout Mice Fed KD

**Figure 6:**
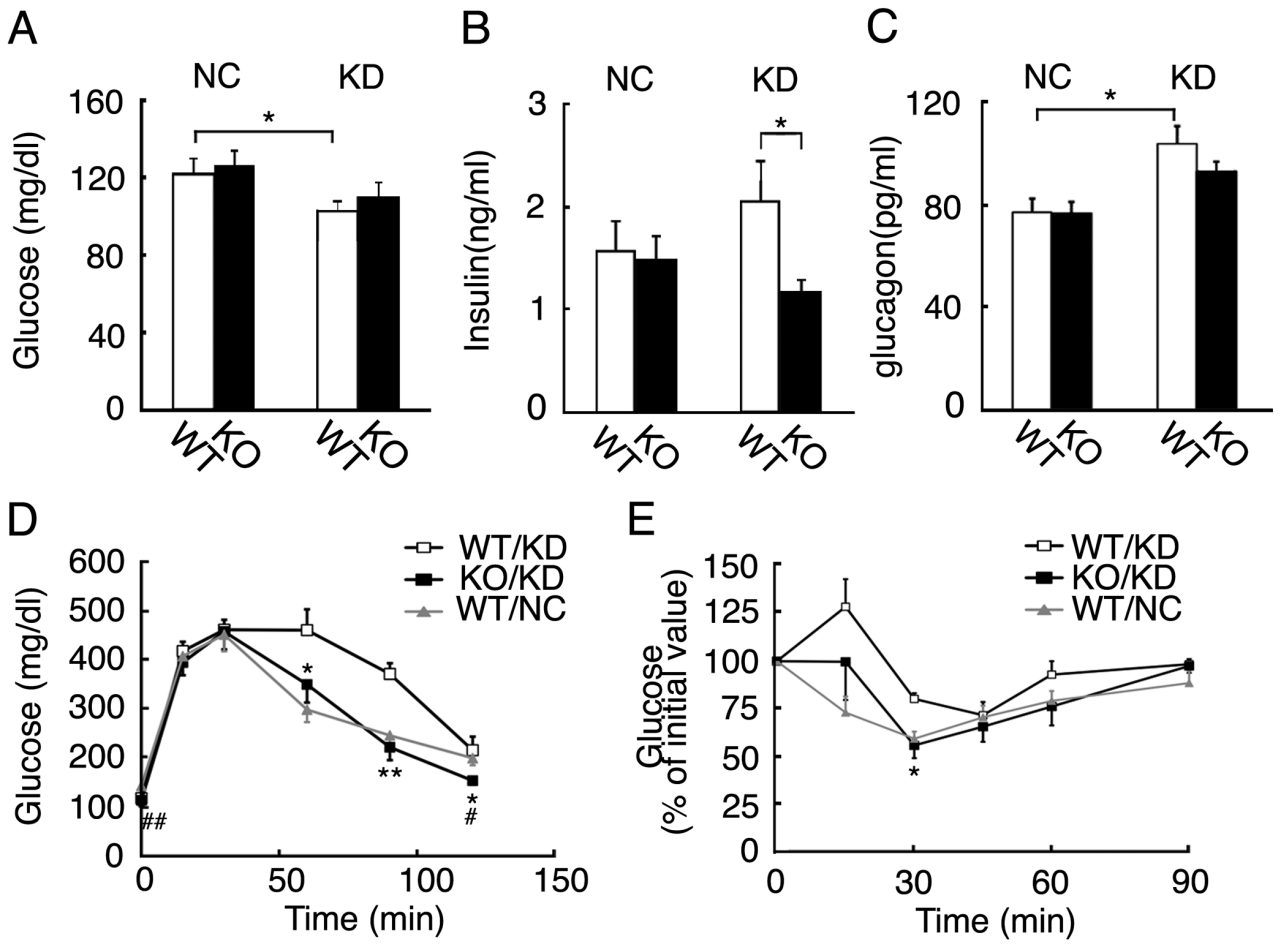
Glucose tolerance and insulin tolerance tests of wild-type and *Fgf21* knockout mice. (A–C) Blood glucose, insulin, and glucagon levels of 3-month-old wild-type (WT) and *Fgf21* knockout (KO) mice fed NC or KD for 6 days (n = 8–12 mice per group; *, *P*<0.05). (D) A glucose tolerance test (GTT) of 3-month-old wild-type (WT) and *Fgf21* knockout (KO) mice fed KD for 6 days (n = 6–10 per group; *, *P*<0.05; **, *P*<0.01 vs. wild-type mice fed KD at the same timepoint, #, *P*<0.05; *P*<0.01 vs. wild-type mice fed NC at the same timepoint). (E) An insulin tolerance test of wild-type (WT) mice fed NC or KD and *Fgf21* knockout (KO) mice fed KD for 6 days (n = 4–7 per group; *, *P*<0.05 vs. wild-type mice fed KD at the same timepoint).

As mice fed KD for 6 days already exhibited impaired insulin sensitivity, resulting in glucose intolerance, we examined blood glucose, insulin, and glucagon levels in wild-type and *Fgf21* knockout mice fed NC or KD for 6 days. Blood glucose levels in both types of mice were slightly but significantly decreased by KD (Figure 6A). Blood glucose levels in the knockout mice were comparable to those in the wild-type mice. Blood insulin levels tended to be increased in the wild-type mice (Figure 6B). However, blood insulin levels in the *Fgf21* knockout mice fed KD were significantly lower than those in wild-type mice. Blood glucagon levels were slightly but significantly increased in wild-type mice fed KD (Figure 6C). However, blood glucagon levels in the *Fgf21* knockout mice fed KD were comparable to those in wild-type mice.

As blood insulin levels were significantly decreased in *Fgf21* knockout mice fed KD, we also examined glucose metabolism in wild-type and *Fgf21* knockout mice fed NC or KD for 6 days using the GTT and ITT (Figure 6D, 6E). The glucose excursion in response to glucose loading during the GTT was significantly decreased in the knockout mice fed KD, compared with that in wild-type mice fed KD. During the ITT, in *Fgf21* knockout mice fed KD, glucose levels remained low at 30 minutes after the insulin loading, compared with those in wild-type mice fed KD.

### 3.7 Insulin-stimulated Akt Phosphorylation in Fgf21 Knockout Mice Fed KD

**Figure 7:**
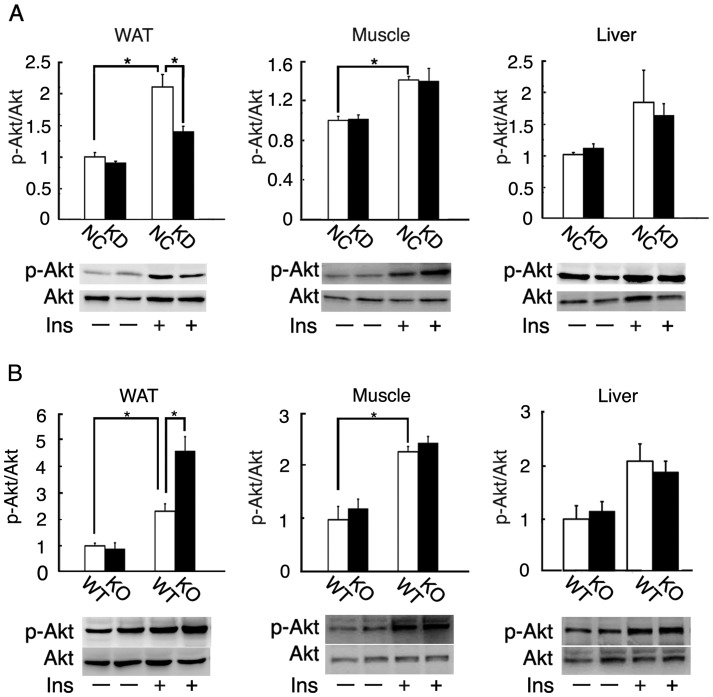
Insulin-stimulated Akt phosphorylation of wild-type and *Fgf21* knockout mice. (A) Basal or insulin-stimulated Akt phosphorylation in the subcutaneous white adipose tissue, gastrocnemius muscle, and liver of 3-month-old wild-type mice fed NC or KD for 6 days (n = 7 per group; *, *P*<0.05). (B) Basal or insulin-stimulated Akt phosphorylation in the subcutaneous white adipose tissue, gastrocnemius muscle, and liver of 3-month-old wild-type (WT) and *Fgf21* knockout (KO) mice fed KD for 6 days (n = 7 per group; *, *P*<0.05). Akt and the phosphorylated form were detected by Western blotting with specific antibodies.

Akt is an obligate mediator of the metabolic actions of insulin, including glucose uptake [18]. To examine which tissues contributed to the insulin resistance, we examined the insulin-stimulated phosphorylation of Akt in the white adipose tissue, gastrocnemius muscle, and liver of wild-type mice fed KD for 6 days (Figure 7A). Insulin-stimulated Akt phosphorylation in the white adipose tissue was significantly decreased in the wild-type mice fed KD for 6 days compared with those fed NC. In contrast, insulin-stimulated Akt phosphorylation in the muscle and liver was unchanged.

To elucidate the roles of Fgf21 in insulin sensitivity in white adipose tissue, we examined the insulin-stimulated phosphorylation of Akt in the white adipose tissue of wild-type and *Fgf21* knockout mice fed KD for 6 days (Figure 7B). Insulin-stimulated Akt phosphorylation in the white adipose tissue was significantly increased in the knockout mice compared with the wild-type mice. In contrast, insulin-stimulated Akt phosphorylation in the muscle and liver was not significantly increased in *Fgf21* knockout mice fed KD.

### 3.8 The Expression of Hepatic Gluconeogenic Genes in Fgf21 Knockout Mice Fed KD

**Figure 8:**
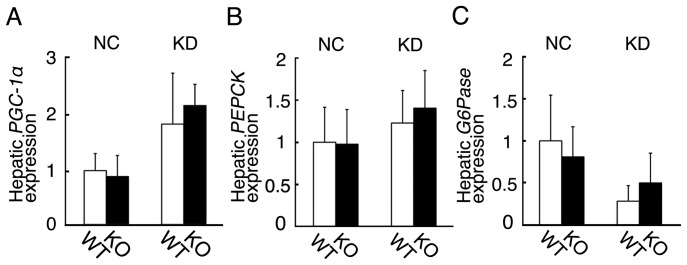
Hepatic expression of key genes involved in gluconeogenesis in wild-type and *Fgf21* knockout mice fed NC or KD. Hepatic expression of *PGC-1*α, *PEPCK*, and *G6Pase* in wild-type (WT) and *Fgf21* knockout (KO) mice fed NC or KD for 6 days. (n = 6–8 mice per group).

In *Fgf21* knockout mice fed KD, glucose levels tended to be lower those in wild-type mice fed KD at the later time points after the insulin loading (Figure 6E). These decrease in glucose levels at the later points potentially suggest that hepatic gluconeogenesis is impaired in the *Fgf21* knockout mice fed KD. Therefore, we examined hepatic expression of three key gluconeogenic genes [19] (Figure 8). The expression levels of *PPARγ coactivator–1α* (*PGC-1α*), *phosphoenolpyruvate carboxykinase* (*PEPCK*), and *glucose-6-phosphatase* (*G6Pase*) in wild-type mice fed KD were unchanged, compared with those fed NC. Those three genes in *Fgf21* knockout mice were essentially comparable to those in wild-type mice. In addition, we examined the blood levels of growth hormone and cortisol, both of which stimulate hepatic gluconeogenesis [20], [21]. The blood levels of growth hormone in wild-type mice were significantly increased by KD feeding. However, its levels in the *Fgf21* knockout mice fed KD were comparable to those in wild-type mice (data not shown). The blood levels of cortisol were essentially unchanged by KD in wild-type mice. Its levels in *Fgf21* knockout mice fed NC or KD were comparable to those in wild-type mice (data not shown).

## Discussion

### 4.1 Effects of KD Feeding on Glucose and Lipid Metabolism

In the present study, we examined glucose and lipid metabolism in mice fed KD for 6 or 14 days. Blood β-hybroxybutyrate levels were significantly increased at 6 days and those at 6 days were comparable to those at 14 days, suggesting that ketogenesis is effectively induced by KD feeding for 6 days. Impairments of glucose tolerance and insulin sensitivity were also effectively induced by KD feeding for 6 days. These results suggest insulin sensitivity to be impaired, probably to maintain blood glucose levels against insufficient amounts of carbohydrate, in mice fed KD. In contrast, blood NEFA and triglyceride levels were unchanged by KD feeding for 14 days. All together, insulin sensitivity was impaired by KD feeding for 6 days, probably independent of changes in body weight and blood lipid levels. In addition, insulin signaling in white adipose tissue was impaired in the mice fed KD, but that in liver and muscle was not. These results suggest that whole-body glucose tolerance in mice fed KD is impaired by the impairment of insulin signaling in white adipose tissue. However, James et al. reported that white adipose tissue accounts for less than 10% of whole-body glucose uptake in rat fed normal chow [22]. Therefore it cannot be assumed that the impairment of glucose uptake in white adipose tissue is solely responsible for whole-body glucose intorelance in mice fed KD. It is possible that whole-body glucose intolerance induced by KD feeding might be caused by the change of glucose metabolism in other tissues including liver and muscle, in addition to the impairment of insulin signaling in white adipose tissue. Alternatively, white adipose tissue might contribute to whole-body glucose uptake much more under a low-carbohydrate malnutritional state than a normal state. Further studies are required to reveal the detailed mechanism underlying the altered glucose metabolism in mice fed KD.

### 4.2 Essentially No Role of Fgf21 in Hepatic Ketogenesis, Triglyceride Metabolism, and Gluconeogenesis in Mice Fed KD

Experiments using *Fgf21* transgenic or Fgf21 protein-administered mice indicate that Fgf21 exerts pharmacological effects on hepatic ketogenesis [7], [9]. However, our previous experiments using *Fgf21* knockout mice fasted for 24 hours demonstrated that Fgf21 is not physiologically required for hepatic ketogenesis [8]. The present experiments using *Fgf21* knockout mice fed KD for 6 or 14 days also show that Fgf21 is not physiologically required for hepatic ketogenesis. These results indicate that the physiological roles of Fgf21 in hepatic ketogenesis are different from its pharmacological effects. Adenoviral knockdown of hepatic *Fgf21* expression in mice fed KD for 4 days resulted in fatty liver and reduced blood β-hybroxybutyrate levels [10]. However, our *Fgf21* knockout mice fed KD did not develop fatty liver (data not shown).

Hepatic gluconeogenesis is known to affect the glucose recovery after the insulin loading during ITT. In *Fgf21* knockout mice fed KD, glucose levels remained low, compared with those in wild-type mice fed KD at the later time points after the insulin loading. Therefore, we examined hepatic gluconeogenic genes expression and blood growth hormone levels in wild-type or *Fgf21* knockout mice fed KD. However, those in the *Fgf21* knockout mice fed KD were comparable to those in wild-type mice, suggesting that Fgf21 is not required for the hepatic gluconeogenesis in the mice fed KD.

### 4.3 Roles of Fgf21 in Insulin Sensitivity in Mice Fed KD

KD feeding for 6 days induced both glucose intolerance and insulin resistance and hepatic *Fgf21* expression. Therefore, we examined the potential roles of Fgf21 in the impairments of glucose tolerance and insulin sensitivity. Although the blood glucose levels in *Fgf21* knockout mice were comparable to those in wild-type mice, the blood insulin levels were significantly decreased. Results of the GTT and ITT showed that glucose intolerance and insulin resistance were significantly improved in *Fgf21* knockout mice. These results indicate that Fgf21 expression induced by KD feeding impairs insulin sensitivity. Furthermore, the phosphorylation of Akt in the white adipose tissue of *Fgf21* knockout mice fed KD was significantly increased, but that in liver and muscle was not. To examine the adipocyte function, we also examined lipolytic activity in white adipocytes in wild-type and *Fgf21* knockout mice fed KD for 6 days. Consistent with the NEFA levels, The lipolytic activity in the white adipose tissue of wild-type mice was essentially unchanged by KD and that in *Fgf21* knockout mice was not significantly increased by KD (data not shown). These results indicate that Fgf21 induces glucose intolerance by impairing adipocyte insulin sensitivity in mice fed KD.

Inflammation in the white adipose tissue is reported to cause insulin resistance [23]. We examined the expression of inflammation-related genes including *Tumor necrosis* factor-α (*Tnf-*α), *Monocyte chemoattractant protein-1* (*Mcp-1*), and *Serine protease inhibitor 2* (*Serpin 2*) in the white adipose tissue of wild-type or *Fgf21* knockout mice fed NC or KD. However, KD feeding did not significantly affect their expression levels (data not shown). These results indicate that insulin resistance induced by Fgf21 is not mediated by inflammation in the white adipose tissue. Therefore, the molecular mechanism underlying the impairment of insulin sensitivity in white adipose tissue remains unknown.

*Fgf21* is also expressed in the white adipose tissue as well as in liver. It was reported that Fgf21 induced to express by cold exposure or rosiglitazone regulated Pparγ activity in the white adipose tissue in an autocrine/paracrine manner, respectively, although it did not affect blood Fgf21 levels [24], [25]. For example, *Fgf21* expression in the white adipose tissue was at least about 5 times higher in cold conditions than in normal conditions [25]. Therefore, we examined *Fgf21* expression in the white adipose tissue of mice fed KD. *Fgf21* expression levels in the white adipose tissue in mice fed KD were significantly but slightly higher than those in mice fed NC and were much lower than hepatic *Fgf21* expression levels (data not shown). These results suggest that hepatic Fgf21, which acts in an endocrine manner, is a negative regulator of adipocyte insulin sensitivity in the adaptation to a low-carbohydrate malnutritional state induced by KD feeding.

### 4.4 Difference between Two Fgf21 Knockout Mouse Lines Fed KD

Badman et al. reported the phenotypes of *Fgf21* knockout mice fed KD for 2 weeks [10]. Their mice had mild weight gain, developed hepatosteatosis, and showed partial impairments of ketogenesis. Furthermore, their mice fed KD for 2 weeks had mild glucose intolerance in the absence of a significant change in insulin sensitivity. The phenotypes of our *Fgf21* knockout mice fed KD for 6 days differed from those of their mice fed KD for 2 weeks. We also examined our knockout mice fed KD for 2 weeks. Our *Fgf21* knockout mice fed KD for 2 weeks did not show mild weight gain, did not show partial impairments of ketogenesis, did not develop hepatosteatosis, and did not develop mild hyperglycemia or hyperinsulinemia (data not shown). Our mice were fed a diet (Harlan TD.96355) consisting of 15.3% protein, 0.6% carbohydrate, and 67.4% fat (wt/wt). The *Fgf21* knockout mice of Badman et al. were fed a diet (Bio-Serv F3666) consisting of 9.5% protein, 0.76% carbohydrate, and 78.9% fat (wt/wt). However, both KDs induced ketogenesis. Blood β-hydroxybutyrate levels in our mice were comparable to those in their mice (0.74±0.07 mmol/L vs. 0.81±0.13 mmol/L, respectively) [10]. In addition, both KDs markedly induced hepatic *Fgf21* expression; therefore, we believe that the difference between the diets did not cause the differences between the two knockout lines. The reason for the difference between the two lines of mice remains unknown.
